# Chemical Analysis of Commercial Functionalized Graphene Nanoplatelets along the Production Process with Raman Spectroscopy and X‑ray Photoelectron Spectroscopy

**DOI:** 10.1021/acs.jpcc.5c06820

**Published:** 2025-12-08

**Authors:** Loay Akmal Madbouly, Heinz Sturm, Alexander Doolin, Vasile-Dan Hodoroaba, Jörg Radnik

**Affiliations:** † Division 6.1 Surface and Thin Film Analysis, 42220 Federal Institute for Materials Research and Testing (BAM), 12203 Berlin, Germany; ‡ 418175 Haydale Limited, Ammanford SA18 3BL, United Kingdom

## Abstract

Commercial applications increasingly rely on functionalized graphene nanoplatelets (GNPs) supplied as powders, aqueous suspensions, and printable inks, yet their process–structure–property relationships across the production chain remain to be fully mapped. Here we apply a correlative Raman spectroscopy (Raman) and X-ray photoelectron spectroscopy (XPS) workflow to nine independent industrial graphene batches spanning three surface chemistries, raw (R), fluorinated (F), and nitrogen-functionalized (N), in all three physical forms which are powders, suspensions, and inks. Raman mapping (with a 532 nm excitation laser) showed that *I*_2D_/*I*_G_ is highest for N-samples and lowest for R-ink. A 2D-vs-G correlation places all samples on a trajectory parallel to the pure-doping vector, which can correlate to holes in the graphene lattice. The mean point-defect spacing is *L*_D_ = 8.4–10.0 nm. High-resolution XPS resolves the accompanying chemical changes: F-powder exhibits distinct C–F (289 eV), C–F_2_ (292 eV), and C–F_3_ (293 eV) components and loses roughly half its F content upon dispersion in deionized water or ink formulation; inks of all chemistries show a pronounced O–C=O peak near 289–290 eV originated from the ink compounds. N-functionalized samples showed a prominent C–N (285.5 eV) only for the ink formulated N-functionalized sample. This study establishes a process-aware blueprint linking the functionalization route and formulation step to lattice disorder and surface chemistry, offering transferable quality-control metrics for graphene supply chains in industrial products/applications such as coatings, storage devices, and printed electronics.

## Graphical abstract

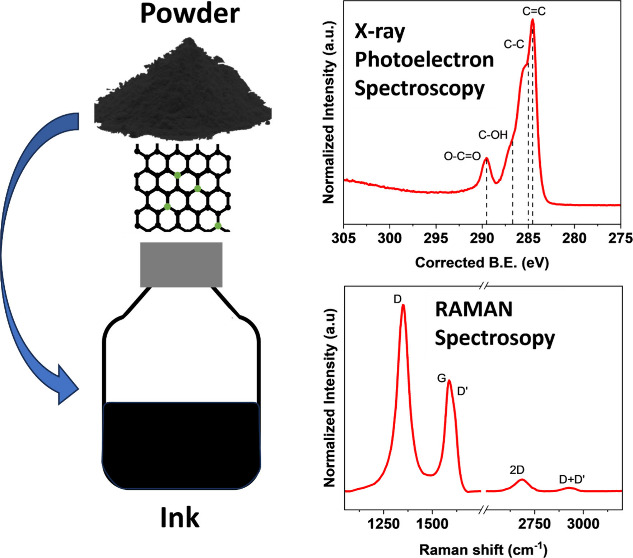

## Introduction

Graphene, a two-dimensional (2D) sp^2^-carbon hexagonal structure^1–3^ with exceptional electrical, mechanical, and thermal properties,^4–8^ underpins a growing class of advanced materials that now permeate inks, coatings and printable electronics at the industrial scale.^9^ Tailoring its surface chemistry through functionalization processes yields functionalized graphene nanoplatelets (GNPs) whose wetting behavior, charge-carrier density and interfacial adhesion can be tuned as needed, attributes that are indispensable for high-throughput manufacturing of storage devices, flexible circuits, and electromagnetic shields.^10,11^ Yet the structure–property relationships across graphene’s production chain, from powders to aqueous suspensions and formulation of inks, remain under-characterized,^12^ mainly because most academic studies focus on exfoliated or chemical-vapor deposition (CVD) graphene prepared under tightly controlled laboratory conditions.^13^

To use commercial graphene reliably, whether in conductive pastes for sensors, barrier coatings, or battery additives, an in-line and production rate compatible, nondestructive analysis^14^ is needed to track how its structure and surface chemistry evolve from the raw powder to the final ink. Raman spectroscopy (Raman) is a strong candidate since a single 532 nm laser scan takes a few seconds, needs no vacuum, and leaves the sample virtually untouched. Graphene’s fingerprints are well-known:^15^ the D-band represents the broken symmetry (edges, vacancies, sp^3^ sites),^16^ the G-band reflects the intact aromatic network,^17^ and the 2D-band responds to both number of layers and charge density.^18^ Because graphene has no band gap,^19^ every visible laser wavelength is resonant (i.e., the virtual energy state which the excited molecule goes to is close to or overlaps a real energy state), so the signals are strong even in disordered, few-layer graphene flakes. Over the past decade, scientists had learned to connect simple features in the spectra such as intensity peak rations of the D-band to the G-band (*I*_D_/*I*_G_) and the 2D-band to the G-band (*I*_2D_/*I*_G_), band positions, and peaks’ widths in order to quantitative parameters such as defect spacing, strain, and doping level.^20–23^ This makes Raman Spectroscopy an ideal tool for rapid structural inspection and charge carrier analysis of graphene. X-ray photoelectron spectroscopy (XPS) provides the complementary quantitative surface analysis.^12,24^ High-resolution XPS (HR-XPS) enables deconvolution of the C 1s envelope into its possible chemical states such as sp^2^, sp^3^, C–O, C–N, and C–F contributions. This holds also true for F 1s and N 1s envelopes for F-functionalized and N-functionalized graphene, respectively. The Raman–XPS combination is uniquely capable of thoroughly investigating the chemistry of functionalized graphene.

Extending on the literature, we provide in this paper Raman–XPS correlative analysis to industrial-grade functionalized GNPs. Specifically, we map the evolution of point-defect density and carrier concentration by statistically evaluating *I*_D_/*I*_G_, *I*_2D_/*I*_G_ and peak dispersions for raw (R-) graphene, fluorine-functionalized (F-) graphene, and nitrogen-functionalized (N-) graphene in all three physical forms; quantify surface stoichiometry and bonding configurations (C–F_*x*_, C–N, carbonyl, carboxyl, etc.) via HR-XPS, linking chemical changes to the Raman spectroscopic markers; and establish a process–structure–property blueprint that rationalizes how commercial plasma functionalization and subsequent compounding of resin and carbon-black influence both lattice integrity and surface chemistry of graphene inks. This work therefore provides a production-scale, multimodal characterization protocol for commercial functionalized graphene, offering a transferable benchmark for quality control in printable-electronics supply chains. It is important to note that, in this manuscript, the term “graphene” is used to refer to industrial graphene, which is composed of carbon structure mixtures namely, GNP and graphite rather than its ISO definition.^25^

## Materials and Methods

In this study, the samples are categorized into three distinct groups based on their form: powders, suspensions, and inks, as shown in Table 1. The powders serve as the primary graphene feedstock for the preparation of suspensions and inks, making their characterization essential to investigate the chemical changes occurring during the production process of graphene inks for industrial applications. Furthermore, the analysis of graphene suspensions is critical, as understanding the dispersion behavior of graphene in aqueous environments provides valuable insights into its biological interactions and toxicological profiles.

**Table 1:** Summary of Labeling of All Nine Materials Used in This Study of Their Physical Forms: Raw Graphene (R), Fluorine-Functionalized Graphene (F), and Nitrogen-Functionalized Graphene (N)

	R	F	N
Powder	R-powder	F-powder	N-powder
Suspension	R-suspension	F-suspension	N-suspension
Ink	R-ink	F-ink	N-ink

Raw graphene powder (R-powder) was functionalized in a proprietary HDPlas plasma reactor (Haydale Ltd., Ammanford, UK).^26^ Fluorine or ammonia gas was supplied to the reaction chamber, depending on the targeted functionalization. The process was carried out in a low-pressure plasma with gas throughput and chamber pressure regulated by mass-flow control and throttled pumping. During treatment the rotating barrel used as the counter-electrode continuously mixed the powder around the central electrode, ensuring uniform exposure and batch homogeneity of both starting material and product. Commercial inks were formulated from 80 wt % diacetone alcohol (resin) as the drying agent and a solvent, 10 wt % carbon black (CB), to provide electrical percolation, pigmentation, and antiagglomeration, and 10 wt % graphite as the precursor to graphene. A separate mixture of 80 wt % resin and 20 wt % CB (resin+CB) was also prepared for XPS analysis.

For Raman analysis, the powders were deposited on a quartz substrate, which was accounted for when processing the Raman spectra. The suspensions were prepared by dispersing the powders in deionized (DI) water followed by gentle shaking, as prepared for commercial applications. The suspensions were then wire-printed,^27^ which is a controlled and optimized form of drop-casting, onto mirror-polished silicon thoroughly precleaned (Si) wafer and dried overnight under low vacuum. Scanning electron microscopy (SEM) was used to ensure a high concentration of deposited material into a spot after wire-printing. The viscous inks were spread directly onto Si wafers, as prepared. For XPS, suspensions and inks were prepared identically to the Raman protocol (the same substrates and deposition steps). For the powders, the samples were gently leveled into an XPS recess with a spatula. All preparation steps followed our protocol described in a previous work.^28^

The Raman spectroscope analysis was carried out, at room temperature, using a custom-made WiTec alpha300 confocal Raman microscope utilizing a 532 nm laser, Peltier-cooled CCD spectrometer, a connecting fiber with 50 μm core, and 20x objective. For the analysis, the spectra were background-subtracted and smoothed. The peaks were fitted with Gaussian curves to determine the height as the intensity, FWHM, and the peak-position. The D/G intensity ratios were indeed taken by assuming the G and D′ as one peak with one intensity.

XPS analysis was performed using an Ulvac-PHI “Quantes” spectrometer (Chanhassen, USA) equipped with an Al Kα source (1486.6 eV). Survey measurements were carried out with a 100 μm beam spot size and a beam power of 25 W, a pass energy of 280 eV, and a step size of 1 eV. High resolution spectra were obtained with sampe spot size and a spotz size, but with a pass energy of 55 eV, and a step size of 0.1 eV. Elemental compositions (in at. %) were quantified from peak areas following Shirley background subtraction, using the relative sensitivity factors supplied by the manufacturer with MultiPak (version 9.9.2) software. The binding energy scale for all graphene samples was calibrated to graphitic carbon at 284.5 eV, while the resin+CB mix was calibrated to aliphatic carbon at 285.0 eV. The spectra were fitted with CasaXPS (version 2.3.1) using Gaussian–Lorentzian curves and Shirley backgrounds.

## Results and Discussion

Raman mapping was performed over nine different batches, followed by spectral smoothing and Gaussian fitting. Powders were mapped over 20 × 20 μm^2^ areas on a 50 × 50-point grid (2 500 spectra per map); suspensions over 30 × 30 μm^2^ on a 30 × 30 grid (900 spectra); and inks over 100 × 100 μm^2^ on a 100 × 100 grid (10 000 spectra). The standard deviation of the Raman peak positions ranged from 0.2 to 0.8 cm^–1^ for the D-bands, from 0.4 to 0.7 cm^–1^ for the G-bands, and from 2 to 13 cm^–1^ for the 2D-bands, for all samples. Figure 1 presents the Raman spectra of the R, F, and N samples, revealing the prominent presence of the D-band, G-band, D′-band, 2D-band, and D+D′-band in all samples. The shape and intensity of the aforementioned bands confirm the presence of the so-called damaged graphene structure.^29^ Raman spectra of a pure graphene structure are characterized by an *I*_2D_/*I*_G_ ratio higher than 2. In all spectra, the G-band and the 2D-band were detected which are the fingerprint for graphene structures.^15^ Despite the low graphene content (<10 wt %) in inks, damaged graphene structure features are detected. G-band represents the E_2_g in-plane vibrational mode of the sp^2^ (hybridized) atoms.^30^ The 2D-band (also called G′-band), on the other hand is sensitive to the number of layers and the amount of doping.^31^ The D-band is activated only when the graphene structure is defective. The strong presence of the D-bands in our results indicated the many structural defects in our commercial graphene samples, unlike monolayer pristine graphene, where the D-band is absent. Figure S1 benchmarks our D-band positions measured at 532 nm (2.33 eV) with the literature data acquired at other excitation energies, showing that our points fall on the expected linear D-mode dispersion (i.e., change of the Raman shift by changing the excitation energy) of approximately 50 cm^–1^ eV^–1^ and validating the spectral calibration.^32^

**Figure 1:**
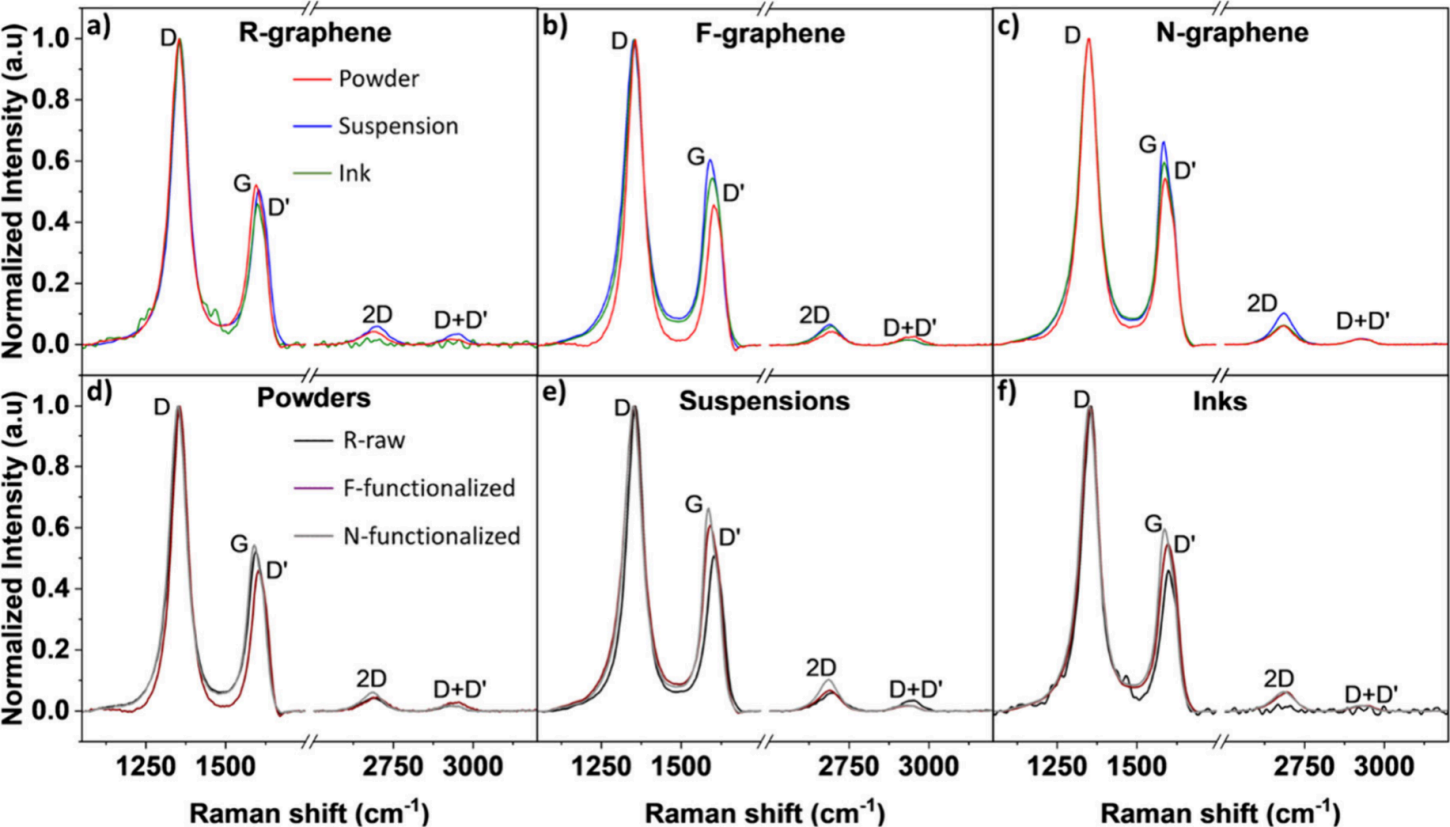
Raman spectra of all powders, suspensions, and inks of a) R-, b) F- and c) N-graphene. The shifts in the G-band regions (1500–1700 cm^–1^) across the samples highlight the structure differences, due to functionalization. The overlaps of the Raman spectra of the R-, F-, and N-surface treated samples in d) powder, e) suspension, and f) ink forms are also presented.

Figure 2 summarizes the peak intensity ratios of the D-bands to the G-bands, *I*_D_/*I*_G_, and 2D-bands to the G-band, *I*_2D_/*I*_G_ for all nine samples. The *I*_D_/*I*_G_ is proportional to the amount of symmetry-breaking defects in the graphene structure.^17,33^ Relative to their raw counterparts, the F- and N-functionalized samples show lower *I*_D_/*I*_G_, in suspensions and inks, suggesting a decrease in the number of defects. In powders, F-functionalization leads to an increase of *I*_D_/*I*_G_, while N-functionalization brings it toward the raw level, indicating that F-functionalization introduces additional Raman-active disorders and N-functionalization has an insignificant effect regarding altering the disorder. *I*_2D_/*I*_G_ provides a valuable insight on the strain and the doping level and nature (holes or electrons) of graphene, since doping decreases the *I*_2D_ significantly and has negligible effect on *I*_G._ ^34^ N-functionalized samples exhibit the largest intensity ratios *I*_2D_/*I*_G_ and thus the lowest residual charge carrier densities, whereas the raw graphene ink (R-ink) shows the smallest ratio, identifying it as the one with the most holes. However, R-ink also shows the highest degree of uncertainty in the *I*_2D_/*I*_G_ analysis. F-functionalization shifts the raw suspension and powder to intermediate, nearly identical *I*_2D_/*I*_G_ values, equaling their carrier densities.

**Figure 2:**
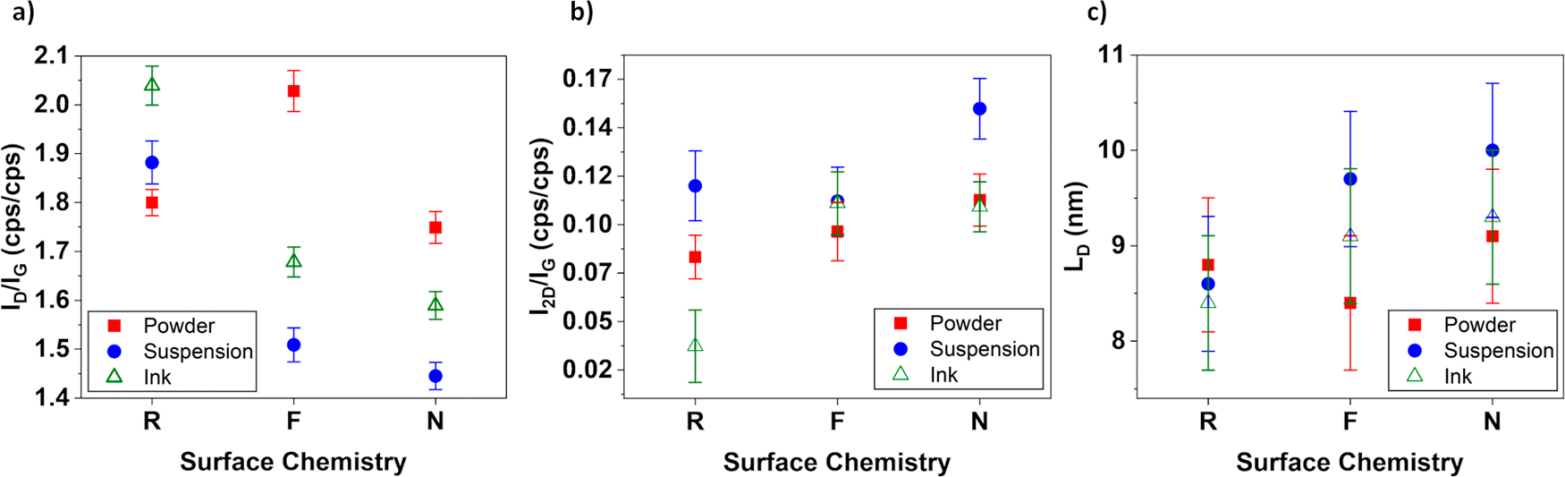
a,b) Raman intensity ratios for three independent commercial graphene forms which are powder (■, red), suspension (●, blue), and ink (△, green), as a function of surface chemistry (R = raw, F = fluorine-functionalized, N = nitrogen-functionalized). In (a), defect indicator *I*_D_/*I*_G_; lower values correspond to larger average defect spacing. In (b), doping indicator *I*_2D_/*I*_G_; higher values reflect lower carrier density. Each point represents a separate batch. In (c), mean point-defect spacings, *L*_D_ (nm), of the nine commercial graphene batches obtained from the Cançado point-defect relation is shown. The error bars combine the propagated random uncertainty from *I*_D_/*I*_G_ with the ±0.7 nm systematic uncertainty as noted in Table S2. The trend confirms that functionalization, in particular nitrogen treatment, widens the average point-defect spacing, while raw inks exhibit the highest defect density.

The mean spacing between symmetry breaking defects (*L*_D_) was calculated with the Cançado relation for 532 nm excitation (Table S2).^33^ Since eight of the nine samples yield *L*_D_ = 8.4–9.8 nm, i.e., only ∼1 nm below the validation threshold proposed by Cançado the values should be regarded as approximate (±0.7 nm systematic plus the propagated random error reported in Table S2). Several independent criteria confirm that all materials, unfunctionalized and functionalized, in different existence state (powder, suspension or ink), remain in the stage-1 point-defect regime:^17^ the defect ratio *I*_D_/*I*_G_ is <2.1; the *I*_2D_/*I*_G_ ratio is ≥0.08 for eight materials (and 0.036 for the single R-ink outlier), well above the 0.05 collapse characteristic of stage-2 disorder;^30^ the G-band widths lie in a narrow 59–68 cm^–1^ band, within 5% of one another, so the broadening is dominated by the spectrometer response (≈57 cm^–1^) and not by extensive defect scattering. The determined values of graphene parameters, collectively, place the materials on the rising branch of the Ferrari–Robertson diagram.^30^ Note that our aim is not to extract an absolute defect density but to compare industrial graphene grades, on a common reference, relative to one another. Within the method’s uncertainty, the data show that F-functionalization enlarges the average defect spacing by ∼1 nm (i.e., powder, 8.4 → 9.3 nm; suspension, 8.8 → 9.8 nm), whereas N-functionalization produces the least-defective as well as the least-doped material in every physical form. These relative shifts, rather than the absolute *L*_D_ numbers, are essential for assessing how commercial processing routes affect the defects of the graphene flakes.

Figure 3 provides a schematic illustration of the possible effect of functionalization on graphene point lattice defects. One panel depicts a graphene domain containing two Raman-active point defects (red), such as vacancies and chemically unpassivated sites, separated by an average distance (*L*_D_) of 8 nm. Upon functionalization (others panel), the sites are chemically modified or electronically passivated (blue), reducing their Raman scattering cross-section and therefore decreasing the measured *I*_D_/*I*_G_ ratio and increasing the *I*_2D_/*I*_G_ ratio. The passivated sites may still be chemically distinct, but their contribution to double-resonant D-band processes is suppressed (i.e., Raman inactive). Note that Figure 3 is only an illustration to explain the change in bands intensities upon functionalization of graphene.

**Figure 3:**
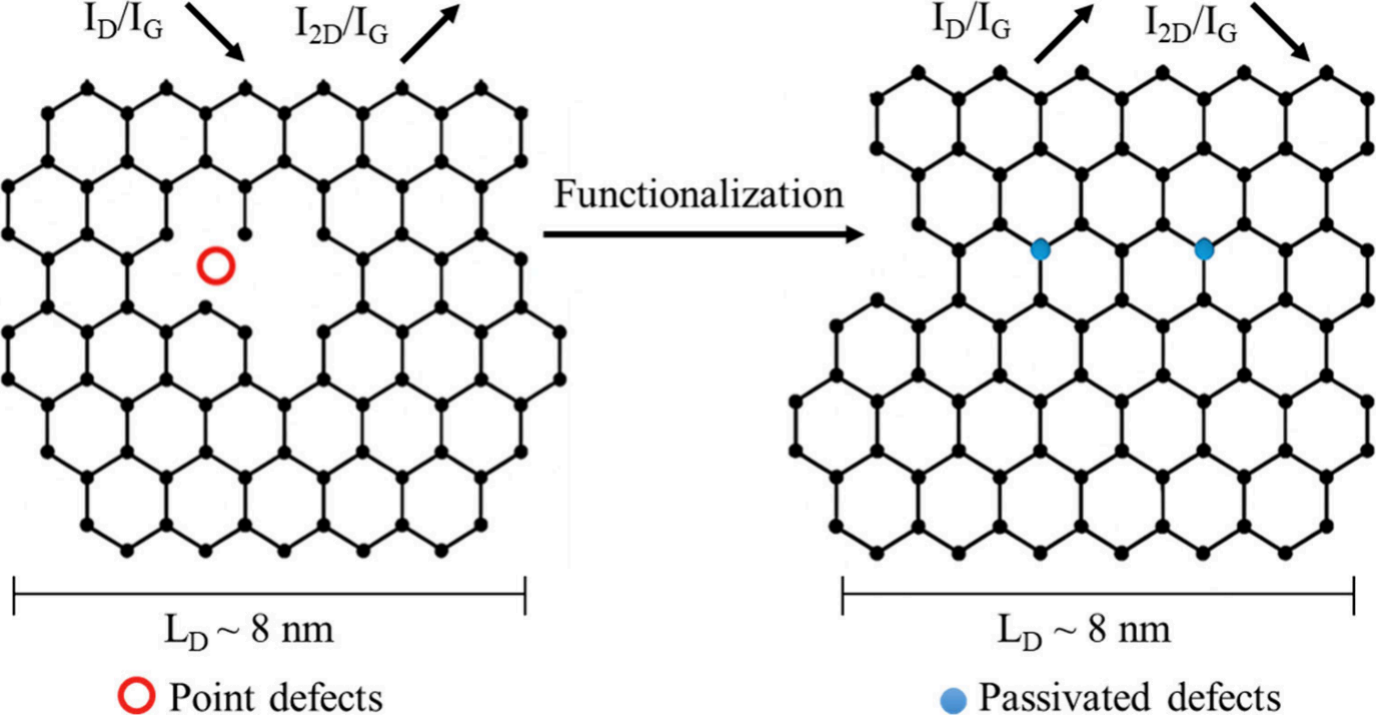
Schematic illustration of the proposed effect of functionalization on defect-related Raman scattering in graphene. Red circles denote Raman-active point defects (e.g., vacancies), while blue dots represent Raman-inactive sites that have been chemically modified or passivated, which reduces the *I*_D_/*I*_G_ ratio.

The positions of the Raman G-bands and 2D-bands of graphene are highly sensitive to a graphene flake’s local environment, shifting in response to doping, strain, interactions with solvents or substrates, and functionalization.^32,35^ Figure 4 shows the correlation between the positions of 2D-bands and G-bands with marker color encoding the Full Width at Half-Maximum (FWHM) of 2D-bands. Two reference trajectories are superimposed: a red dashed-line (slope 2.2) for charge-neutral graphene under randomly oriented uniaxial strain and a dashed green line (slope of 0.7) for doping.^36^ In the case of increase of hole-doping levels, the G- and 2D-bands upshift over the doping line.^34^

**Figure 4:**
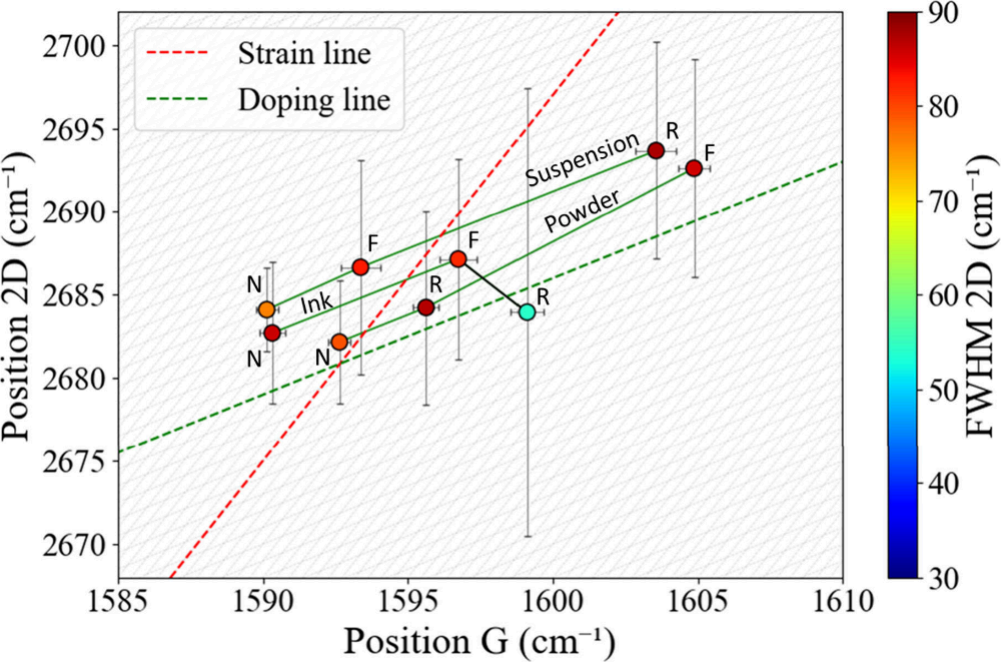
Correlation between the 2D-band and G-band peak positions for the nine graphene samples with their relative degrees of confidence for both Raman bands. Each marker represents one sample; the marker color encodes the 2D-band FWHM according to the adjacent color bar.

All nine data points fall on a line parallel to the doping line, indicating that the hole concentration is the main variable across the series and not the strain. For N-graphene samples, fewer holes could be detected than for the R-suspension and the F-powder. The scatter across the doping line implies a similar residual strain in all samples. R-ink deviates from the common trend but also exhibits the largest error bars, highlighting the batch-to-batch variability of raw commercial graphene inks and supporting the industrial practice of chemical functionalization of graphene to give more control over its properties.

While Raman spectroscopy reveals how functionalization modifies the lattice vibrationally, it provides limited information regarding the qualitative and quantitative states of the elements in the samples. To identify those species and quantify their surface concentrations, we now consider XPS and HR-XPS.

Table 2 shows the atomic fraction ratios of the atomic fractions of the O/C, F/C, and N/C for the R-, F-, and N-graphene materials, respectively. Those ratios are obtained from the XPS elemental survey spectra, where the elemental composition of each sample is reported in at. %. The results indicate that oxygen content in the R-graphene samples increased from powder to suspension to ink. It also indicates the fluorine content decreased by half in the suspension and ink forms compared to the powder form, while the nitrogen content remained virtually the same across all N-graphene forms. These results and their interpretation are found in the literature^28^ where energy dispersive X-rays (EDX), XPS and SEM were utilized to characterize them.

**Table 2:** Atom Fraction (at. %) Ratios O/C for R-Graphene (R), F/C Ratio for F-Graphene (F) and N/C for N-Graphene (N) Reported from Our Previous Work on the Same Materials^28^

	**R**	**F**	**N**
	**O/C**	**F/C**	**N/C**
**Powder**	0.19	0.32	0.03
**Suspension**	0.21	0.16	0.03
**Ink**	0.25	0.16	0.02

The overlap of high-resolution XPS C 1s spectra in Figure 5a–c for all nine materials reveals the change of the chemical state of carbon across different forms: powders, suspensions, and inks. Figure 5a shows the HR-XPS C 1s spectra of all R-graphene materials in which powders and suspensions show similar trends, while R-ink deviates by showing a shoulder at ∼285.5 eV corresponding to nitrogenated carbon and another prominent peak at 289 eV corresponding to the carboxyl group. These two peaks are prominent across all ink samples, as shown in Figure 5f.

**Figure 5:**
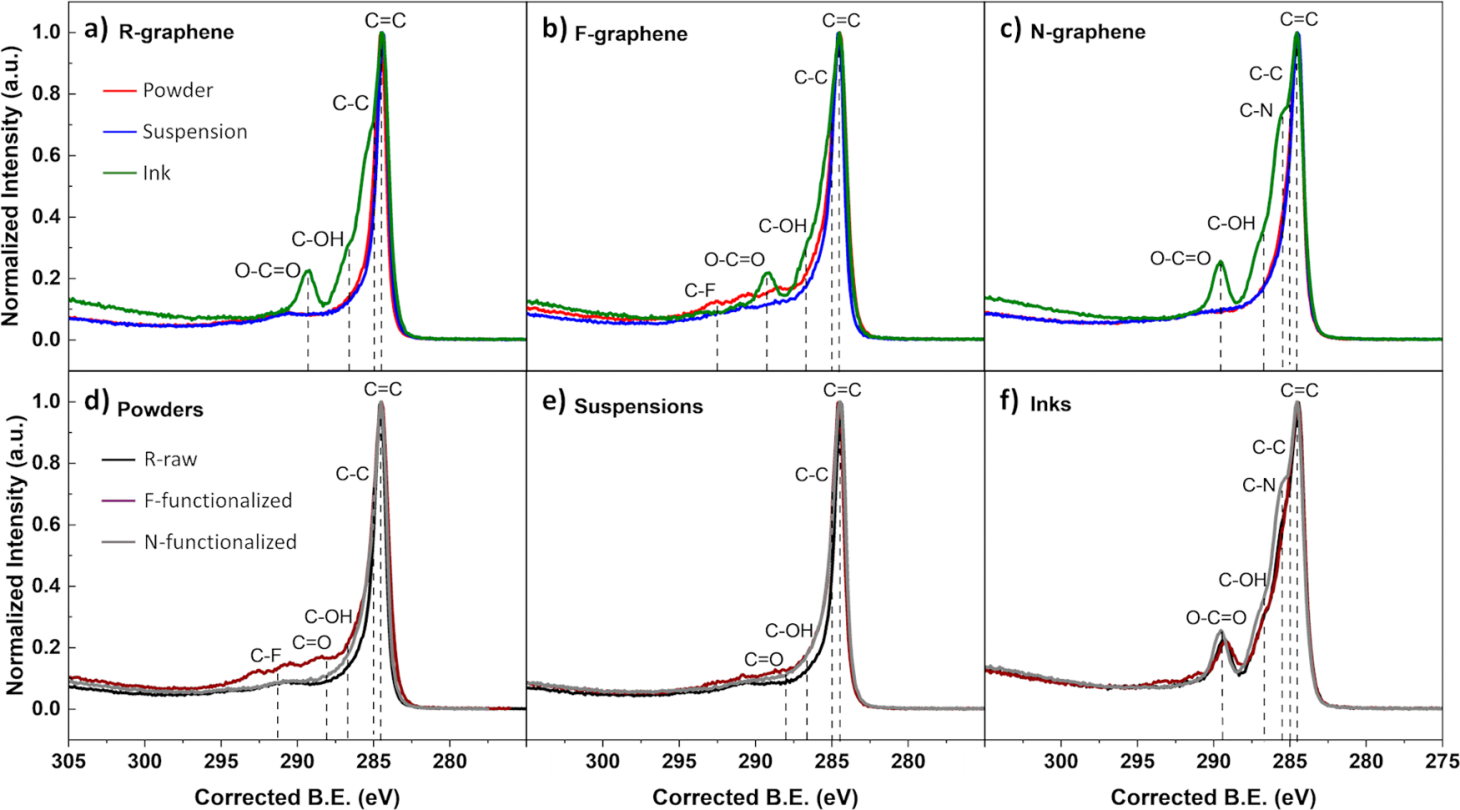
XPS spectra of C 1s state of a) unfunctionalized graphene (R), b) F-functionalized graphene (F), and c) N-functionalized graphene (N) as powder, in suspension and in the inks, as well as the overlapped spectra of the differently functionalized samples raw, F- and N-functionalized as d) powder, e) in suspension, and f) in the ink..

In Figure 5b, the F-suspension’s signal drops below the F-powder’s intensity starting at 287 eV, consistent with partial loss of C–F functionality upon dispersion in DI water or resin+CB, as shown in Table 2. F-powder exhibits in Figure 5d three distinct fluorocarbon peaks at 289 eV (C–F), 292 eV (C–F_2_) and 293 eV (C–F_3_).^37^ The nitrogenated carbon shoulder at ∼285.5 eV in the N-powder spectrum likely arises from C–N/C–O overlap, preventing accurate deconvolution. In the suspensions (Figure 5e), all functionalized samples show enhanced intensity above 285 eV, reflecting increased oxygen content possibly due to sp^3^ C–C bond breaking and subsequent hydroxyl/carbonyl formation during the functionalization process. Finally, the inks (Figure 5f), constituting only ∼10 wt % graphene, display a pronounced carboxyl (O–C=O) peak at ∼289 eV, and the N-ink features a small shoulder at 285.5 eV attributable to C–N (also contributed by the ∼1 at. % N in the resin+CB matrix). The deconvolution of C 1s of R-, F- and N-inks can be found in Supporting Information Table S1 and Figure S2.

All identified binding energies, obtained from literature,^38^ are tabulated in Table 3. The fittings of HR-XPS C 1s spectra for all samples are listed in Figure S2 in the Supporting Information.

**Table 3:** Binding Energies Used in the HR-XPS Analysis in This Study as Obtained from the Literature^38^

**Chemical assignment**	**Binding energy (eV)**
**C=C**	284.5
**C–C**	285.0
**C–OH**	286.5
**C=O**	288.0
**O–C=O**	∼289
**C–N**	∼286
**C–F**	287.7
**C–F**_**2**_	∼292
**C–F**_**3**_	∼293
**Graphitic Nitrogen**	∼401
**Organic Fluorine**	689.0

HR-XPS analysis of the F 1s core electrons (Figure 6a) confirms the presence of organic fluorine and the absence of metallic fluorine, which is typically observed at 684 eV, across all F-functionalized graphene samples. The N 1s spectra in Figure 6b reveals a major peak for the N-ink (above 400 eV) which is attributable to graphitic or hydrogenated N, compared to the N-powder and N-suspension with a further peak below 400 eV which is attributable to pyrrolic N.^39^

**Figure 6:**
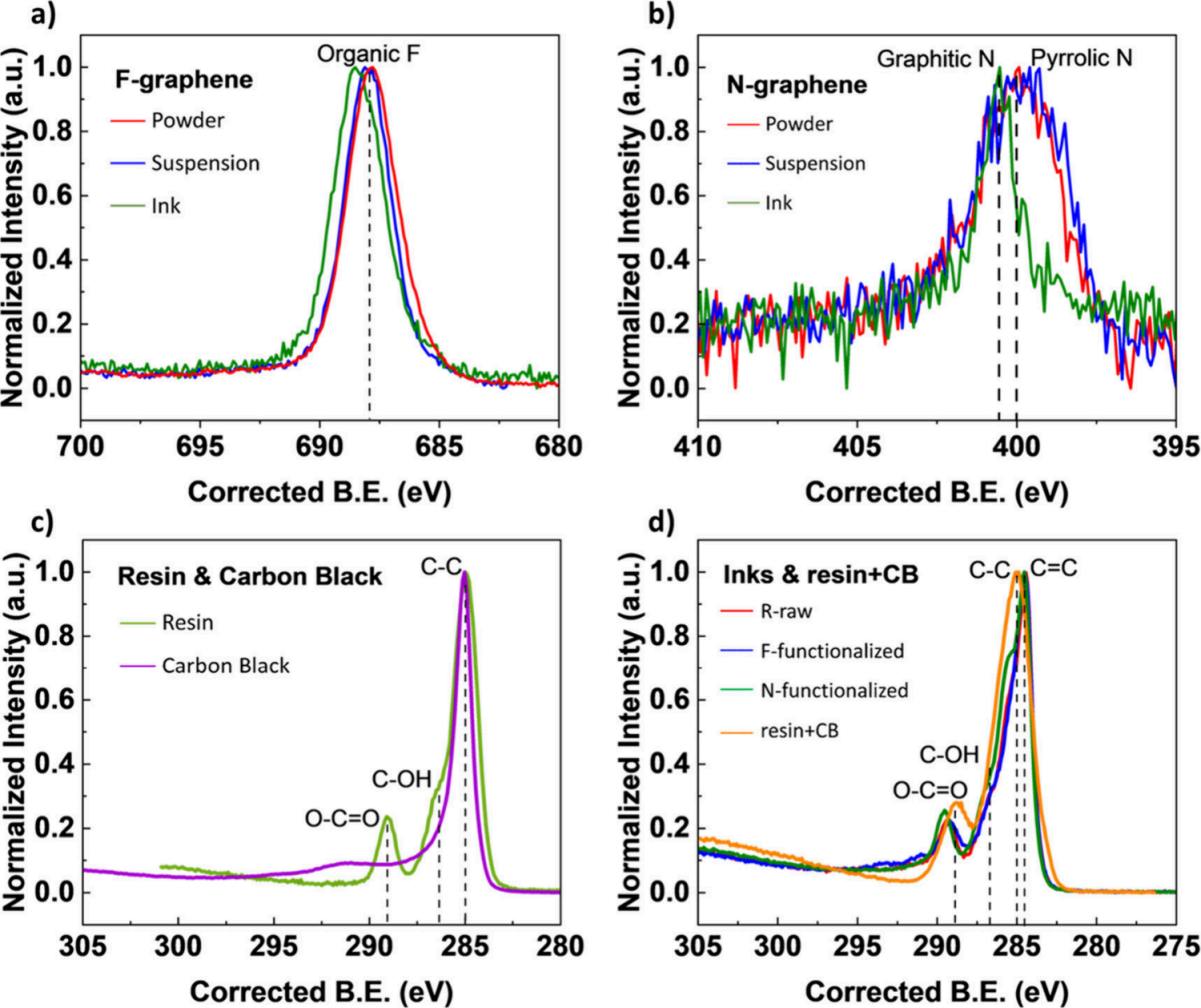
Overlap of HR-XPS spectra of a) F 1s for all F-functionalized materials in all forms, b) N 1s spectra for N-functionalized samples in all forms, and C 1s for c) resin and CB, and d) all inks with the resin+CB mix.

The HR-XPS C 1s spectrum of CB does not show any peak at carboxyl or carbonyl binding energy; diacetone as a component in the ink shows a prominent peak at the exact binding energy (Figure 6c). Figure 6d shows the overlaying of C 1s high-resolution spectra of the resin+CB mixture and the spectra of the inks. The O–C=O peak is observed with a slight shift in the resin+CB mix.

## Conclusions and Outlook

We established a correlative Raman–XPS analysis workflow to track commercial graphene from raw powders to suspensions and inks across raw (R), fluorinated (F), and nitrogen-functionalized (N) surface chemistries. Raman mapping (532 nm) places all nine independent materials in the “stage-1” regime e.g., *I*_D_/*I*_G_ = 1.4–2.0, visible 2D-bands. A 2D-vs-G correlation aligns with the pure-doping trajectory, indicating comparable residual strain, while carrier density varies with chemistry/form. Using the Cançado calibration, the point-defect spacing is *L*_D_ ≈ 8.4–10.0 nm; values near the 10 nm boundary are reported with propagated and systematic uncertainties and interpreted comparatively. XPS investigates the following chemistry: F-powder shows distinct C–F, C–F_2_, C–F_3_ components and loses F upon dispersion in water or ink formulation; all inks exhibit a pronounced O–C=O feature near 289–290 eV; N-ink shows a more significant C–N contribution from the C 1s spectra compared to N-powder and N-suspensions. Together, these markers link the functionalization route and formulation to lattice disorder and surface chemistry.

Practically, we propose production-compatible metrics: *I*_D_/*I*_G_, *I*_2D_/*I*_G_, the 2D–G trend using Raman spectroscopy, and C 1s, F 1s, and N 1s HR-XPS analysis with O/C, F/C, and N/C at. % ratios. Within this analysis framework, N-functionalization yields the least-doped, least-defective graphene samples across all forms, whereas raw inks are the most p-doped and variable within the nine commercial materials considered in this study. Future work should extend these readouts to in-/at-line monitoring under realistic coating and printing conditions.

## Supplementary Material



## Notes

D-band dispersion benchmarking (Figure S1); HR-XPS C 1s fitting parameters (Table S1); HR-XPS C 1s peak-fitting spectra for R-, F-, and N-inks (Figure S2); Raman-derived defect metrics and uncertainty model, including *I*_D_/*I*_G_ statistics and interdefect spacing LD (Table S2) (PDF)
