# Supplementary material for: Chemical Analysis of Commercial Functionalized Graphene Nanoplatelets along the Production Process with Raman Spectroscopy and X‑ray Photoelectron Spectroscopy
Source: J Phys Chem C Nanomater Interfaces. 2025 Dec 8;129(50):22033–40. doi: 10.1021/acs.jpcc.5c06820 (PMC12720484; doi:10.1021/acs.jpcc.5c06820)
Supplement: Supplementary file 1 [file jp5c06820_si_001.pdf]

Chemical Analysis of Commercial Functionalized Graphene Nanoplatelets along the Production process with Raman spectroscopy and X-ray photoelectron spectroscopy

Loay Akmal Madbouly[1], Heinz Sturm[1], Alexander Doolin[2], Vasile-Dan Hodoroaba[1,*], Jörg Radnik[1,*]

[1] Federal Institute for Materials Research and Testing (BAM), Division 6.1 Surface and Thin Film Analysis, Berlin, Germany

[2] Haydale Limited, Ammanford, United Kingdom

Correspondence
Vasile-Dan Hodoroaba, Federal Institute for Materials Research and Testing (BAM), 12200 Berlin, Germany.
Email: dan.hodoroaba@bam.de
Jörg Radnik, Federal Institute for Materials Research and Testing (BAM), 12200 Berlin, Germany.
Email: joerg.radnik@bam.de

Keywords: functionalized graphene, commercial graphene, graphene inks, Raman spectroscopy, X-ray photoelectron spectroscopy

---

Table of Content:

Figure S1: The D-bands positions versus the excitation energy of all the graphene samples used in this study (red) where the Raman measurements were taken with 532 nm (2.33 eV) laser, compared to the graphitic/graphene Raman spectra (black) obtained from literature (1). The D-band positions of the nine D-bands found in this study fall on the established dispersion (dashed) line, confirming the calibration and peak assignment.

Table S1: Fitting parameters of the HR-XPS spectra of the C 1s photoelectrons used in this study. GL(30) is 30% Lorentzian and 70% Gaussian. LA(1.2,2.1,5) is Lorentzian with asymmetry parameter of 1.2, asymmetry decay rate of 2.1 and a width parameter of 5.

Figure S2: The HR-XPS of C 1s photoelectrons peak fittings of samples a) R-, b) F-, and c) N-inks.

Table S2: Raman-derived defect metrics and uncertainty model. Reported are the peak-height ratios I_D/I_G (mean over each map) and their standard deviations (STD), together with the inter-defect spacing L_D (nm) computed from the Cançado relation for 532 nm excitation, so that C′ (532 nm) ~1.44×10^2 nm^2.

Section 1: Benchmarking the D-band dispersion.

Figure S1 benchmarks the D-band positions found in this study at 532 nm (2.33 eV) against literature data acquired at other excitation energies [1]: the literature points (black) with their corresponding linear regression line (dashed-line). Note that the linear regression line follows the known linear D-mode dispersion, with slope of ~50 cm^{-1} eV^{-1}, and our nine values (red) at 2.33 eV lie on this trend within the experimental scatter, confirming our spectral calibration.

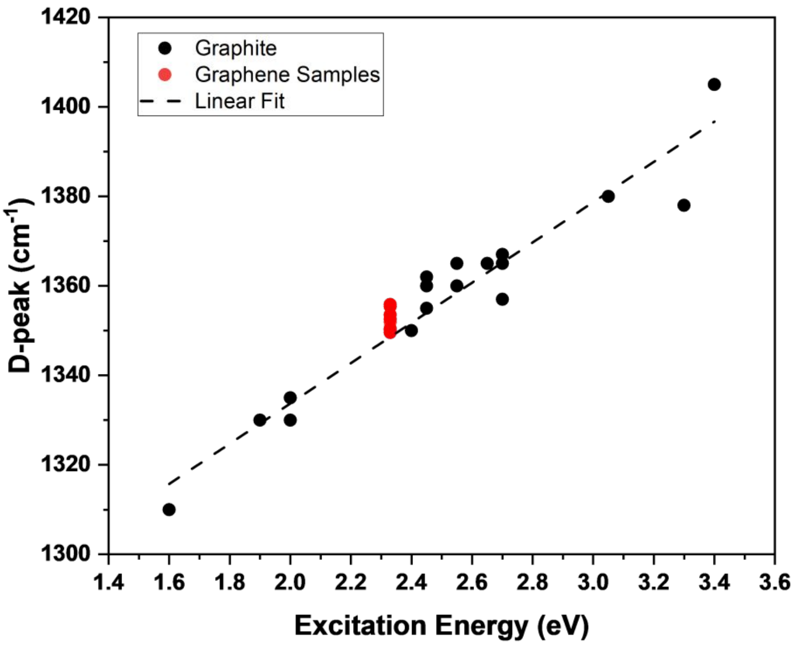

Figure S1. The D-bands positions versus the excitation energy of all the graphene samples used in this study (red) where the Raman measurements were taken with 532 nm (2.33 eV) laser, compared to the graphitic/graphene Raman spectra (black) obtained from literature [1]. The D-band positions of the nine D-bands found in this study fall on the established dispersion (dashed) line, confirming the calibration and peak assignment.

Section 2: Deconvolution of high resolution XPS (HR-XPS) C 1s spectra for all materials

The spectra were corrected to the C1s peak of C=C at 284.5 eV. The fitting parameters including Full-Width-at-Half-Maximum (FWHM), line shape and common position (ranges) for each peak are tabulated in Table S1.

Table S1. Fitting parameters of the HR-XPS spectra of the C 1s photoelectrons used in this study. GL(30) is 30% Lorentzian and 70% Gaussian. LA(1.2,2.1,5) is Lorentzian with asymmetry parameter of 1.2, asymmetry decay rate of 2.1 and a width parameter of 5.

<table>
<tr><th>Chemical Assignment</th><th>Position (Range) /eV</th><th>FWHM /eV</th><th>Line shape</th></tr>
<tr><td>C=C</td><td>284.5</td><td>0.8 to 1.1</td><td>LA(1.2,2.1,5)</td></tr>
<tr><td>C-C</td><td>284.7 to 285.0</td><td rowspan="7">0.9 to 1.5</td><td rowspan="8">GL(30)</td></tr>
<tr><td>C-OH</td><td>286.3 to 286.7</td></tr>
<tr><td>C=O</td><td>287.7 to 288</td></tr>
<tr><td>C-O</td><td>~286.0</td></tr>
<tr><td>C-N</td><td>~285.5</td></tr>
<tr><td>O-C=O</td><td>288.8 to 289.3</td></tr>
<tr><td>C-F</td><td>~ 291</td></tr>
<tr><td>π-π*</td><td>290.9</td><td>2.7</td></tr>
</table>

Figure S2 shows the deconvolution of the HR-XPS spectra of the C 1s photoelectrons of all graphene materials used in this in this study.

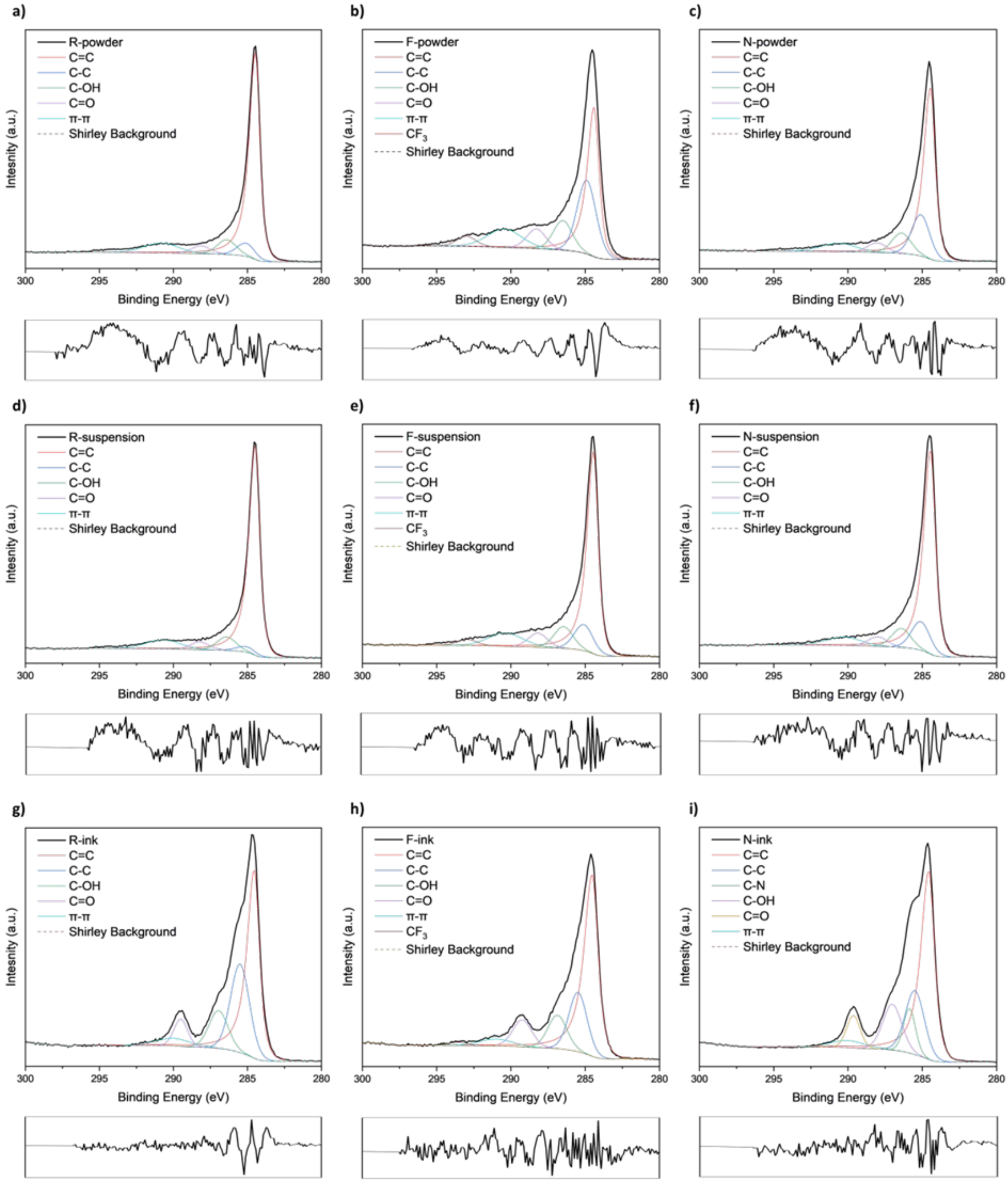

Figure S2. The HR-XPS of C 1s photoelectrons peak fittings, with their relative residuals, of samples R-, F-, and N in (a-c) powder, (d-f) suspension, and (g-i) ink forms.

Section 3:

The Tuinstra–Koenig C(λ) constant is an empirical constant that relates I_D/I_G to the crystallite size L_a in polycrystalline graphite and multilayered graphene structures with "stage-1" defects [2]. Its value is dependent on the wavelength of the excitation laser. Cançado *et al.* derived a more precise relation that keeps the same λ^4 dependence, but switches to L_D^2 (i.e., the mean spacing between any symmetry-breaking defect. In this study, L_D was calculated using Cançado method (Figure 2c), taking $C'_{(532)} = 1.8\times10^{-9} \times \lambda^4 \approx 1.4\times10^2$ nm² (Eq.1):

$$L_D = \sqrt{\frac{(1.4 \times 10^2)}{I_D/I_G}}$$

The uncertainties in Figure 2 in the main manuscript were calculated using the propagation of uncertainty formula (Eq. 2):

$$\sigma_{X/Y} = \frac{X}{Y} \cdot \sqrt{\left(\sigma_X/X\right)^2 + \left(\sigma_Y/Y\right)^2}$$

Table S2. Raman-derived defect metrics and uncertainty model. Reported are the peak-height ratios I_D/I_G (mean over each map) and their standard deviations (STD), together with the inter-defect spacing L_D (nm) computed from the Cançado relation for 532 nm excitation, so that C' (532 nm) ~1.44×10^2 nm². Random uncertainties in L_D were propagated from the measured I_D/I_G spread using first-order error propagation and combined in quadrature with the systematic calibration uncertainty of ± 0.7 nm reported by Cançado et al. *to give the total L_D error. The analysis assumes the stage-1 defect regime for which the Cançado model is valid. (These uncertainties are the ones used for the L_D error bars in Figure 2.)*

				**L_D (uncertainty)**		
	**I_D/I_G**	**I_D/I_G (STD)**	**L_D**	**Random**	**Systematic**	**Total**
	cps	cps	nm	nm	nm	nm
R-Powder	1.80	0.03	8.95	0.07		0.70
R-Suspension	1.88	0.04	8.75	0.10		0.71
R-Ink	2.04	0.04	8.41	0.08		0.71
F-Powder	2.03	0.04	8.43	0.09		0.71
F-Suspension	1.51	0.03	9.78	0.11	0.7	0.71
F-Ink	1.68	0.03	9.27	0.10		0.71
N-Powder	1.75	0.02	9.08	0.06		0.70
N-Suspension	1.45	0.02	9.99	0.07		0.70
N-Ink	1.59	0.02	9.52	0.07		0.70

The systematic error reported by Cançado is ± 0.7 nm. The L_D (Random Error) obtained from the I_D/I_G STD was calculated using Error Propagation (Standard First Order) (Eq. 3)

$$\sigma_{L_D}^{rand} = \frac{1}{2} \frac{L_D}{I_D/I_G} \sigma_{I_D/I_G}$$

Since the random (measurement) and systematic (calibration) uncertainties are independent, they were quadratically added to one another (Eq. 4):

$$\sigma_{L_D}^{total} = \sqrt{\sigma_{L_D}^{rand\,2} + \sigma_{L_D}^{sys\,2}}$$

References

(1) Ferrari, A. C.; Basko, D. M. Raman spectroscopy as a versatile tool for studying the properties of graphene. *Nat Nanotechnol* **2013**, *8* (4), 235–246. DOI: 10.1038/nnano.2013.46

(2) Tuinstra, F.; Koenig, J. L. Raman Spectrum of Graphite. *The Journal of Chemical Physics* **1970**, *53* (3), 1126–1130. DOI: 10.1063/1.1674108
